# Reply: Statins for the prevention of cirrhosis complications: An American emulation of the StatLiver trial

**DOI:** 10.1097/HC9.0000000000000551

**Published:** 2024-10-30

**Authors:** Nina Kimer, Thit M. Kronborg, Flemming Bendtsen

**Affiliations:** Gastro Unit, Medical Division, Copenhagen University Hospital Hvidovre, Hvidovre, Denmark

We thank Dr Tapper and colleagues for their excellent emulation of the StatLiver trial,^1^ in which we compared atorvastatin to a placebo to prevent the progression of liver disease. Atorvastatin was found to be without beneficial effects; however, the trial was terminated early. In the trial emulation, Tapper and colleagues intended to extend the follow-up with a larger sample size from a nationally representative database of adult Americans. The results conform with the initial trial on the clinical endpoints.^2^

In the trial emulation, 400 people with liver cirrhosis were included. All were stable patients from outpatient clinics, and they differed from the StatLiver trial in that they had fewer hospital admissions and fewer episodes of decompensation. In the Staliver trial, all had portal hypertension, and the majority had ascites.

Prior studies of statins have shown a protective effect against the progression of liver disease, both in mortality and the development of decompensation, but the majority have been retrospective or registry-based.^3^ The Baveno VII guidelines recommend statins for people with cirrhosis with a concomitant indication for statins, as it may have a beneficial effect on survival and portal hypertension.^4^ The StatLiver trial and the trial emulation could not support this statement, neither in patients with mild or advanced liver cirrhosis.

However, there are still areas where further evidence may reveal the potential of statins in cirrhosis, and further studies on the risk of HCC and early stages of cirrhosis development may be of interest in the future,^5^ where long-term follow-up may be key to understanding the possible efficacy of statins in cirrhosis.

The biomolecular mechanisms of statins in the liver are still poorly understood.^1^ Further studies on these areas may identify a window for therapy to benefit patients with or at risk of developing liver cirrhosis.
